# Impacts of Australian Firefighters’ On-Call Work Arrangements on the Sleep of Partners

**DOI:** 10.3390/clockssleep2010005

**Published:** 2020-01-30

**Authors:** Grace E. Vincent, Simone Karan, Jessica Paterson, Amy C. Reynolds, Michelle Dominiak, Sally A. Ferguson

**Affiliations:** 1 Central Queensland University, Appleton Institute of Behavioural Science, Adelaide, SA 5034, Australia; simonemkaran@gmail.com (S.K.); jessica.paterson@cqu.edu.au (J.P.); a.reynolds@cqu.edu.au (A.C.R.); michelle.dominiak@cqumail.com (M.D.); sally.ferguson@cqu.edu.au (S.A.F.); 2 Central Queensland University, School of Health, Medical, and Applied Sciences, Adelaide, SA 5034, Australia

**Keywords:** on-call, partners, sleep, impacts, sleepiness, relationship happiness

## Abstract

On-call work arrangements are commonly utilised in the emergency services sector and are consistency associated with inadequate sleep. Despite sleep being a common shared behaviour, studies are yet to assess the impact of on-call work on the sleep of co-sleeping partners. This study aimed to investigate whether frequent 24/7 on-call work impacted the sleep and relationship happiness of firefighters’ partners. Two key research questions were investigated: (1) Does the frequency of calls impact sleep and relationship happiness? and, (2) Does the (a) sleep quantity and (b) sleep quality of partners impact perceived relationship happiness? A cross-sectional study was conducted using an online questionnaire completed by partners of on-call workers (*n* = 66; 93% female). The questionnaire included items on (i) sleep quantity and quality, (ii) on-call sleep disturbances and, (iii) relationship happiness. Responses were analysed using logistic regression models. Higher overnight call frequency was associated with greater self-reported levels of inadequate sleep (<7 h per night; *p* = 0.024). Support for continuance of a firefighter’s role was less likely if the partner reported they regularly had trouble falling asleep within 30 min (*p* < 0.001). There were no other significant relationships between the frequency of calls or other sleep quantity or quality variables and relationship happiness. This study provides important first insights into how firefighters’ on-call work arrangements impact partners’ sleep. Future research is needed across periods of high and low call demand, using objective measures of sleep to further define the impacts of on-call work on partners’ sleep.

## 1. Introduction

Worldwide, between 17% and 25% of the workforce are involved in some form of on-call working arrangements in order to provide continuity of services [1,2,3]. On-call arrangements in sectors such as emergency services, healthcare and transport are used to cover emergency or unpredictable events, or when workloads may be low, particularly at night [4]. While there are a number of benefits to on-call working arrangements, there are number of potentially adverse consequences for workers [5,6,7,8]. Previous research has shown that when on-call at night, workers’ sleep is often disturbed by the call to attend work [4,9], and may be disturbed even in the absence of receiving a call such that simply being on-call can be detrimental to sleep [10,11,12,13]. Of concern, a recent study reported that 80% of Australian on-call workers obtained less than the recommended 7 h of sleep during on-call periods, at an estimated public health cost of $137 million (AUD) [14]. 

Given the majority of on-call workers attempt to sleep during overnight on-call periods, and sleep is often a shared behaviour [15], the call to work has the potential to wake not only the worker but also their bed partner. In Australia, 14% of the general population report that sleep disturbances due to the behaviour of their partner negatively affects their relationship [16]. Further, studies have reported impairments to daytime functioning and poorer relationship satisfaction when sleep is inadequate [17,18,19]. Therefore, inadequate sleep may also adversely impact the quality of the relationship between the on-call worker and their partner [20]. This in turn may expose both the on-call worker and their bed partner at risk of the adverse consequences associated with inadequate sleep, such as impaired performance, increase likelihood of accidents and long-term health impacts [21,22].

One group of on-call workers that have received little attention are auxiliary (retained) firefighters in Australia. Auxiliary firefighters in Australia provide permanent coverage for emergency situations. These firefighters can be on-call 24 hours a day, 7 days a week (often 365 days a year) and are usually deployed to attend incidents from an off-site location (e.g., their home), often in arduous and physically demanding circumstances [23,24,25]. Sleep of emergency service personnel is primarily impacted by work arrangements [26,27] and environmental conditions [28,29,30]. Further, the few studies addressing sleep in on-call firefighters support these findings with self-reports of poorer sleep among retained firefighters when on-call [31]. Other research has reported that firefighters listed sleep disturbance as the most concerning factor for both workers and partners [32]. Firefighters also reported that due to inadequate sleep they felt more fatigued and sleepy, that inadequate sleep interfered with their personal life, and that they were concerned how on-call work arrangements impacted their partner [32]. Further, given the high-risk nature of the firefighting profession, partners ‘worry’ may be associated with disturbed sleep, as has been shown in previous research [33]. Despite these reports, to date there has been no exploration of perceived sleep disturbance in partners of on-call firefighters, and/or investigation into the associations with relationship happiness and support for the worker’s work arrangements. 

In summary, while it is known that on-call work arrangements impact the sleep of on-call workers [4,9] calls may also impact the sleep of co-sleeping partners as a result of the disturbance of the call and the partner leaving the bed, or as a result of worry for the partner [34]. Partners are also a critical component of on-call workers coping network [35,36], so it is important to also understand challenges they may face. Therefore, this study aimed to investigate whether frequent 24/7 on-call work impacted the sleep and relationship happiness of firefighters’ partners. We explored two key research questions: (1) does the frequency of calls impact sleep and relationship happiness? and (2) does the (a) sleep quantity and (b) sleep quality of partners impact perceived relationship happiness?

## 2. Materials and Methods

### 2.1. Procedures

Participants were provided with a link to an anonymous online survey which included the study information sheet, and an invitation to participate. Prior to commencing the survey, interested participants provided informed consent by completing a declaration confirming that they were an Australian resident, 18 years or older and they understood the information provided. 

### 2.2. Participants and Recruitment

Firefighters were contacted via email listings, and social media networks (e.g., personal and industry-based Facebook pages) within the Queensland Fire and Emergency Services (QFES) and asked to pass the survey link on to their co-sleeping partners for participation in the study. The inclusion criteria were: (1) Australian resident, over 18 years of age, and current co-sleeping partner of a QFES auxiliary firefighter and (2) in the past month participants had not undertaken duties that would disrupt overnight sleep (e.g., shift work). A total of 83 participants started the survey and met the inclusion criteria. Of these, 23 participants only provided demographic information before exiting the survey, resulting in a final sample of 60 participants. 

### 2.3. Measures

The online survey was designed and distributed using a survey tool (Survey Monkey) from June–August 2018. Benefits of using online surveys for data collection include convenience, flexibility, access to participants, cost-effectiveness, and participant anonymity [37]. The research was approved by the CQUniversity Human Research Ethics Committee (approval date: 7 June 2018; approval reference: 2018-036).

### 2.4. Demographic Questions 

The survey included sociodemographic questions about age, sex, employment status, number of dependent children, and relationship length (while partner employed as a firefighter). Questions on the presence of pre-existing conditions that may interfere with sleep were also included. The questions were mostly closed-ended and forced choice style. A free response section was provided at the end of the survey to allow participants to describe their experience as a partner of an on-call auxiliary firefighter. 

### 2.5. Sleep Quantity and Quality

Participants’ sleep quantity and quality were assessed using the Pittsburgh Sleep Quality Index (PSQI) [38]. The PSQI is a widely used sleep measure and provides a retrospective assessment of sleep quality over the previous month [39]. The PSQI assesses the elements of sleep considered to influence overall sleep quality including average sleep, sleep latency, sleep disturbances, self-perception of sleep quality and daytime functioning [38]. Component scores are generated from the items and each component’s score is summed to provide a global score for sleep quality, with higher scores indicating poorer sleep quality [38]. The seven components used to calculate the global PQSI have a high internal consistency, with overall Cronbach’s alpha coefficient of 0.83 [38]. The PSQI also has good overall internal stable test-retest reliability and good criterion and convergent validity [40]. The global score for the PSQI was used to separate individuals into categories of sleeper quality, with individuals who scored a global PSQI of less than or equal to 5 categorised as ‘good quality sleepers’ and those with a global PSQI score of greater than 5 as ‘poor quality sleepers’ [38]. 

### 2.6. On-Call Sleep Disturbances

To assess the impact of overnight on-call activity, questions were included on (a) sleep due to the call alarm, (b) the return of the worker from a call, and (c) partner’s worry about the worker out on a call. These additional questions followed the same 4-point Likert response criteria included in the PSQI (i.e., ‘not during the last month’ (0), ‘less than once a week’ (1), ‘once or twice a week’ (2), and ‘three or more times a week’ (3)). Questions on a self-reported assessment of sleep quality were also included to gauge perceptions of partner sleep when calls did and did not occur. Questions about the ability of the partner to return to sleep after being woken by a call and when a worker returns from a call were also included to assess impacts of on-call activity on sleep of the partner. To assess how on-call sleep disturbances impact the quality of sleep, questions on subjective sleep quality when calls do and do not occur were also included. 

### 2.7. Relationship Happiness

Relationship happiness was assessed using a single-item proxy variable from the Dyadic Adjustment Scale (DAS-7) [41]. The question assesses relationship happiness by asking about the degree of relationship happiness on a 7-point Likert scale ranging from ‘extremely unhappy’ (0) to ‘perfect’ (6). 

### 2.8. Statistical Analysis

Responses to each ordinal scale were coded according to the research question. For relationship happiness, for instance, the primary interest was estimating the odds of reporting a ‘happy’ relationship or above. Thus, a response in any of the categories, including ‘happy’, ‘very happy’, ‘extremely happy’, and ‘perfect’, was coded as ‘1’; and all other responses (i.e., ‘extremely unhappy’, ‘fairly unhappy’, or ‘a little unhappy’) as ‘0’. The binary coding for responses to other questions is as follows:

- Partner’s firefighting role (‘very much like’/’somewhat want’ my partner to give up role/’neutral’, and ‘somewhat’/’very much like my partner to continue’);
- Rating of sleep when partner is (a) not called and (b) is called (‘very poor’/’rather poor’ and ‘neither poor nor good’/’rather good’/’very good’);
- Ease of returning to sleep a) following a call and b) when partner returns from callout (‘strongly agree’/’agree’/’neither agree nor disagree’ and ‘disagree’/’strongly disagree’);
- Time taken to fall asleep (<30 min or >30 min) and;
- Hours of sleep (<7 h and >7 h).

The only variable not converted to a binary variable was overnight call frequency.

Statistical analyses were conducted using R [42]. For each research question, two logistic regression models were fitted. For binary outcome variables, to determine the impact of an independent variable of interest, we fitted and compared two logic models, one with and the other without the independent variable, referred to as the ‘full model’ and the ‘reduced model’, respectively. Independent variables of interest were frequent 24/7 on-call work, sleep quantity or sleep quality. Both full and reduced models included age, sex, and sleep disorder as covariates. The two models were then compared using a likelihood ratio test to determine whether the effect of the independent variable (RQ 1—frequency of calls, and RQ2—sleep quantity and quality) was statistically significant from zero. Significant impact of an independent variable was declared when the full model had a better fit than the reduced model. The test statistic for the model comparison, −2*log (likelihood ratio), is assumed to follow a chi-square distribution with one degree of freedom. For each continuous outcome, we regressed the outcome on the independent variable of interest with age, sex and sleep disorder as covariates, and a t-test was used to determine whether the effect of the independent variable of interest was significantly different from zero.

## 3. Results

### 3.1. Demographics

Demographic and relationship happiness data are summarised in Table 1. The sample was predominantly female (93%) with a large proportion of participants (63%) aged 35 years or older (mean ± SD; 38.4 ± 10.1). The majority of participants were employed (83%) with the highest proportion working full-time (40%). Most households (61%) were supporting at least one child under 18 years of age. The majority of participants had been in a relationship with a firefighter for an average of 9.5 ± 8.8 years. Most participants (71%) reported their partner received 5 or less calls per month. The majority (92%) of participants were happy in their relationship and only a small proportion (8%) reported that they would like their partner to give up their role as a firefighter. Partners’ self-reported sleep quantity and quality is summarised in Table 2. 

### 3.2. Sleep Related to Calls

The majority of partners (80%) rated their sleep as ‘rather good’ or ‘very good’ when their partner was not called whereas the majority rated their sleep as ‘rather poor’ or ‘very poor’ (61%) when their partner received a call. Most participants (63%) indicated they could not go back to sleep easily when their partner received a call, and around half (53%) were able to go back to sleep easily when their partner returned home from a call. On average participants took 23.3 ± 13.2 min to fall asleep, with 40% falling asleep within 15 min. 

### 3.3. Habitual Sleep

**Figure 1:**
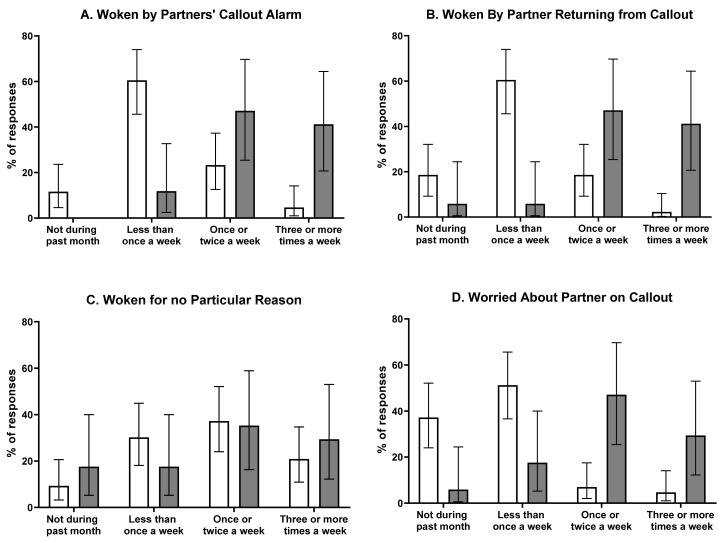
Reasons participants had trouble sleeping. Figures represent % of participants with 95% confidence interval. 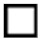 = participants whose partners received ≤ 5 calls per month; 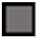 = participants whose partners received >5 calls per month. Please note: *n* = 1 “other” response not shown due to low frequency.

The average amount of sleep per night was 7.3 ± 1.1 h with most (76%) reporting they obtained at least 7 h of sleep per night. Most participants (73%) rated their normal sleep quality during the previous month as ‘fairly good’ or ‘rather good’. The average global PSQI score was 6.7 ± 3.3, with 40% of partners’ habitual sleep quality categorised as good (PSQI ≤5) and 60% of partners’ sleep quality categorised as poor (PSQI >5). Figure 1 summarises the reasons participants had trouble sleeping. 

### 3.4. Research Questions

RQ1. Does the frequency of calls impact sleep and relationship happiness?

The odds of partners obtaining inadequate sleep (<7 h) increased per call by a multiplicative factor of 1.198 (95% CI = [1.007, 1.42]; *p* = 0.024). Therefore, the odds of reporting sleep <7 h for individuals who receive 5 call outs are 1.198 times greater those who receive 4 call outs. Those who receive 6 calls outs have 1.435 (1.198 × 1.198) greater odds of reporting inadequate sleep than those who receive 4 call outs. There were no other significant associations found between frequency of calls and other sleep variables, or relationship happiness.

RQ2. Does the (a) sleep quantity and (b) sleep quality of partners impact perceived relationship happiness?

The odds of partners showing support for their partner’s firefighter role was 93% less among participants who have trouble falling asleep within 30 min, compared with those who fell asleep within 30 minutes (OR = 0.073, 95% CI = [0.014, 0.38]; *p* < 0.001). There were no other significant associations found between partners’ sleep quantity and quality, and relationship happiness.

## 4. Discussion

This is the first study to examine how regular 24/7 on-call work impacts the sleep and relationship happiness of the co-sleeping partners of on-call workers. Partners reported that their sleep was disturbed directly by the callout alarm, the firefighter returning from a callout, and from worrying about the firefighter on callout. Higher overnight call frequency was associated with greater self-reported levels of inadequate sleep (<7 h), however there was no association between overnight call frequency and any other measures of sleep or relationship happiness. 

The current study provides evidence that partners’ sleep is disturbed due to frequent 24/7 on-call work arrangements. Regardless of overnight call frequency (≤5 calls per month or >5 calls per month), over three quarters of participants reported sleep being disturbed directly by the callout alarm, the firefighters returning from a callout, and from worrying about the firefighter on callout. Not surprisingly, the greater the frequency of calls (>5 calls a month) the higher the proportion of responses relating to the frequency of being woken by the callout alarm, returning from a callout, or being worried about a partner during a callout. Previous research has reported that the unpredictability of calls, along with the length and type of callout, are stressful elements of on-call work arrangements for both workers and partners [43]. In the current study, participants note that unpredictability plays a role in their stress, which may in turn impact sleep. For example, “the unpredictable nature of (him) being on-call is very hard... not knowing how long he will be away while on a callout is difficult and can be very worrisome.” Further, most participants (63%) indicated they could not go back to sleep easily when their partner received a call, but around half (53%) were able to go back to sleep easily when their partner returned from a callout. As worry is strongly associated with disturbed sleep [33], the latter finding may be related to a reduction in worry when the firefighter returns safely from a callout. For example, a comment from one participant was “...(I) don’t sleep well until he returns, and I know he is okay”.

There were no relationships observed between the frequency of calls, and sleep quality, ability to go back to sleep after a call, ability to go back to sleep after firefighter returns from a call, bed and wake times, or time taken to fall asleep. However, the odds of a partner obtaining inadequate sleep (<7 h) increased as overnight call frequency increased. In other words, the higher the frequency of calls, the greater the likelihood that a partner reported inadequate sleep. Previous studies provide strong evidence for a link between inadequate sleep and adverse health outcomes (e.g., obesity, dyslipidemia, hypertension) [44,45]. Further, a meta-analysis showed that less than 7 h of sleep per night was associated with increased mortality risk (RR 1.12, 95% CI [1.06–1.18]) [46]. The data for the current study was collected during a ‘low’ demand period of firefighter activity in Queensland (June-August). Therefore, it is possible that the findings of the current study are underestimated, relative to times where work demands are higher (December-February). Future studies should implement longitudinal designs to capture seasonal variations in work demands, to better assess the potential impact of on-call work arrangements on the sleep of workers and their partners.

The only significant finding related to sleep and relationship happiness, was that support for continuance of a firefighter’s role was less likely if participants reported that they regularly had trouble falling asleep within 30 minutes. There were no other relationships between the frequency of calls or other sleep quantity/quality variables, and relationship happiness. The latter finding was particularly unexpected as previous research has reported a positive association between sleep quality and relationship quality [19,39]. An explanation for these findings may be that the majority (92%) of participants were happy in their relationship, and very few participants reported that they would like their partner to give up their role as a firefighter (8%). Further, as the majority of participants had been together for over 5 years (52%), an adaptation to or acceptance of the firefighters’ on-call arrangements may explain the lack of findings related to disturbed sleep and relationship happiness. This is reflected in this quote from a participant, “when we were first together, I used to wake up and stay awake until she returned home, but after 9 years I have learnt not to worry about it so much.” 

Most participants in this study were female (93%), which is not surprising given 90% of QFES firefighters are male, and the majority of relationships in Australia are heterosexual [47]. The majority of participants (77%) reported obtaining an average of >7 h of sleep, higher than the 55% in the general population in Australia reporting >7 h [16]. When sleep quality was assessed using the PSQI, only 40% of partners’ sleep quality was ‘good’ (PSQI ≤5) and 60% of partners’ sleep quality was ‘poor’ (PSQI >5). Further, while most partners (80%) reported their sleep as ‘rather good’ or ‘very good’ when their partner was not called, 61% rated their sleep as ‘rather poor’ or ‘very poor’ when their partner was called. It should be noted that the PSQI assesses sleep quality over the previous month. Thus, these questions do not distinguish between partners’ sleep on call and no-call night, rather these findings reflect partners general sleep quality.

There are a number of implications of the current study for partners of on-call workers, the on-call workers themselves, and for organisations who utilise frequent 24/7 on-call scheduling. The findings suggest that partners’ sleep is disturbed due to firefighters receiving calls and/or being out on call outs. This also means that the overall health, safety and economic impacts of 24/7 on-call scheduling could be largely underestimated. While not assessed in the current study there is also potential for on-call sleep disturbances to extend to other individuals in the household, as many of the participants had young children that may share the sleep environment of their parents [48]. Workplace policies and procedures may need to consider the potential for broader impacts of on-call arrangements on both workers and their families when developing risk management plans and strategies. Such plans may include providing education to workers regarding the potential for their night on-call activity to impact their family, and offering support to workers on how to manage and minimise the potential disruptions and negative impacts of on-call work on their own and their families’ sleep.

This study provides important insight into the impact of on-call work on partners sleep and relationship happiness, however, some limitations should be acknowledged. First, this study was cross-sectional and therefore causality cannot be implied. For example, sleep quantity and quality cannot be directly attributed to on-call sleep disturbances, and other factors known to impact sleep (e.g., existing health conditions) may play a role. Second, the sample size (*n* = 60) was small and limited to the state of Queensland, Australia. Consequently, there is potential for Type II error, and the potential for underestimating the impact of on-call arrangements on firefighting partners’ sleep and happiness. Future studies should utilise a larger sample which may be more representative of the on-call workforce. Third, partners were asked to retrospectively evaluate their sleep based on recall of their average experience. It is possible that this recall, as well as the proximity to their partners’ last call, may have impacted their reported sleep behaviours. Forth, the relationship happiness question employed in this survey was a single item proxy. Future research should include the full version of the Dyadic Adjustment Scale or another validated relationship happiness assessment tool. Fifth, additional questions in regards to partners personality, socioeconomic status/financial needs, strength of support network, length of time needed to adjust to a partners schedule and type of call outs could have enabled further understanding of on-call arrangements impact partners sleep and relationship happiness. Further, while self-report measures are inexpensive, discrete and easy to apply, the discrepancy over the subjectivity of reporting sleep via an online questionnaire, compared to objective measures of sleep should be noted. Future studies should consider polysomnography and actigraphy as valid and objective measures of partners sleep in on-call scenarios. 

## 5. Conclusions

This novel study provides preliminary insights into the impact of on-call work arrangements on the sleep of co-sleeping partners. Higher overnight call frequency was associated with greater self-reported levels of inadequate sleep in partners. Overall, there was no association between frequency of calls or other sleep quantity/quality variables, and relationship happiness. The current findings provide valuable insights that may assist in the development of workplace strategies that help minimise the potentially deleterious aspects of on-call activity on sleep and relationship happiness.

## Figures and Tables

**Table 1:** Partner demographics and relationship happiness.

	Total	
Participant Characteristic	*n*	%
*n*	60	100
		
Sex		
Female	56	**93.3**
Male	4	6.7
		
How old are you? Age group (y)		
18–24 years	5	8.3
25–34 years	17	28.4
35–44 years	20	**33.3**
45–54 years	13	21.7
>55 years	5	8.3
		
How long (in years) have you and your partner been a couple, while your partner has been on-call as a firefighter?		
0–5 years	29	**48.3**
6–10 years	12	20.0
11–15 years	7	11.7
16–20 years	6	10.0
Over 20 years	6	10.0
		
Approximately how many overnight firefighter calls did your partner receive over the past month?		
0–5	43	**71.7**
6–10	13	21.7
11–15	2	3.3
16–20	2	3.3
		
The following represent different degrees of happiness in your relationship. The middle point, “happy”, represents the degree of happiness of most relationships. Please select the degree of happiness, all things considered, in your relationship.		

Extremely/Fairly/A Little Unhappy	5	8.3
Happy/Very/Extremely Happy or Perfect	55	**91.7**
		
With regard to your partner’s auxiliary firefighter role, how do you feel about their participation?		
I would like/very much like my partner to give up role	5	8.3
Neutral	10	16.7
I would like/very much like my partner to continue role	45	**75.0**

**Table 2:** Sleep quality and quantity of partners.

	Total	
Question	*n*	%
Do you have any of the following diagnosed sleep-related disorders or conditions that impact your sleep? (select all that apply)		
No, I do not currently have a diagnosed sleep disorder or condition impacting sleep	41	**68.3**
Snoring	7	11.7
Insomnia	5	8.3
Restless Legs	4	6.7
Sleep Apnoea	0	0.0
Depression	5	8.3
Anxiety	5	8.3
PTSD	0	0.0
Other	4	6.7
		
In general, how would you rate your sleep on a night when your partner is not called?		
Very Poor/Rather Poor	3	5.0
Neither Poor Nor Good	9	15.0
Very Good/Rather Good	48	**80.0**
		
In general, how would you rate your sleep on a night when your partner is called?		
Very Poor/Rather Poor	37	**61.7**
Neither Poor Nor Good	7	11.7
Very Good/Rather Good	16	26.6
		
In general, when I am woken by my partner’s call I can easily go back to sleep.		
Strongly Disagree/Disagree	38	**63.3**
Neither Agree nor Disagree	9	15.0
Strongly Agree/Agree	13	21.7
		
In general, when my partner returns from a call, I can easily go back to sleep if I have the opportunity to do so.		
Strongly Disagree/Disagree	14	23.3
Neither Agree nor Disagree	14	23.3
Strongly Agree/Agree	32	**53.4**
		
During the past month, with regard to sleeping arrangements with your partner, did you usually?		
Sleep with partner in same bed	54	**90.0**
Sleep in another room	6	10.0
		
During the past month, what time have you usually gone to bed on a weekday?		
08:00 pm–08:59 pm	8	13.4
09:00 pm–09:59 pm	26	**43.3**
10:00 pm–10:59 pm	21	35.0
11:00 pm–11:59 pm	4	6.6
12:00 am–12:59 am	1	1.7
		
During the past month, what time have you usually gone to bed on the weekend?		
07:00 pm–07:59 pm	1	1.7
08:00 pm–08:59 pm	2	3.3
09:00 pm–09:59 pm	15	25.0
10:00 pm–10:59 pm	31	**51.7**
11:00 pm–11:59 pm	8	13.3
12:00 am–12:59 am	2	3.3
01:00 am–01:59 am	1	1.7
		
During the past month, how long (in minutes) has it usually taken you to fall asleep when you go to bed?		
1–15 min	24	**40.0**
16–30 min	23	38.3
31–45 min	10	16.7
>45 min	3	5.0
		
During the past month, what time have you usually gotten up in the morning on a weekday?		
04:00 am–04:59 am	2	3.3
05:00 am–05:59 am	17	28.4
06:00 am–06:59 am	26	**43.3**
07:00 am–07:59 am	13	21.7
08:00 am–08:59 am	2	3.3
		
During the past month, what time have you usually gotten up in the morning on the weekend?		
05:00 am–05:59 am	1	1.7
06:00 am–06:59 am	17	28.3
07:00 am–07:59 am	20	**33.3**
08:00 am–08:59 am	12	20.0
09:00 am–09:59 am	7	11.7
10:00 am–10:59 am	2	3.3
11:00 am–11:59 am	1	1.7
		
During the past month, how many hours of actual sleep did you get at night, on average?		
5–5.5 h	7	11.7
6–6.5 h	7	11.7
7–7.5 h	26	**43.3**
8–8.5 h	16	26.7
9–9.5 h	4	6.6
		
During the past month, how would you rate your sleep quality overall?		
Very bad	1	1.7
Fairly bad	15	25.0
Fairly good	38	**63.3**
Very good	6	10.0
		
During the past month, how often have you taken medicine (prescribed or “over the counter”) to help you sleep?		
Not during the past month	50	**83.4**
Less than once a week	2	3.3
Once or twice a week	2	3.3
Three or more times a week	6	10.0
		
During the past month, how often have you had trouble staying awake while driving, eating meals, or engaging in social activity?		
Not during the past month	48	**80.0**
Less than once a week	9	15.0
Once or twice a week	3	5.0
Three or more times a week	0	0.0

## Abbreviations

DAS-7	Dyadic Adjustment Scale
DOAJ	Directory of open access journals
MDPI	Multidisciplinary Digital Publishing Institute
PSQI	Pittsburgh Sleep Quality Index
QFES	Queensland Fire and Emergency Services
RQ	Research question
